# A Concise Review on the Utilization of Abbreviated Protocol Breast MRI over Full Diagnostic Protocol in Breast Cancer Detection

**DOI:** 10.1155/2022/8705531

**Published:** 2022-04-28

**Authors:** Haytham Al Ewaidat, Mohammad Ayasrah

**Affiliations:** ^1^Department of Allied Medical Sciences-Radiologic Technology, Faculty of Applied Medical Sciences, Jordan University of Science and Technology, Jordan; ^2^Jordan University of Science and Technology, Department of Allied Medical Sciences-Radiologic Technology, Faculty of Applied Medical Sciences, Jordan University of Science and Technology, Jordan

## Abstract

Breast MRI possesses high sensitivity for detecting breast cancer among the current clinical modalities and is an indispensable imaging practice. Breast MRI comprises diffusion-weighted imaging, ultrafast, and T2 weighted and T1 weighted CE (contrast-enhanced) imaging that may be utilized for improving the characterization of the lesions. This multimodal evaluation of breast lesions enables outstanding discrimination between the malignant and benign and malignant lesions. The expanding indications of breast MRI confirm the far superiority of MRI in preoperative staging, especially in the estimation of tumour size and identifying tumour foci in the contralateral and ipsilateral breast. Recent studies depicted that experts can meritoriously utilize this tool for improving breast cancer surgery despite their existence of no significant long term outcomes. For managing the, directly and indirectly, associated screening cost, abbreviated protocols are found to be more beneficial. Further, in some of the patients who were treated with neoadjuvant chemotherapy, breast MRI is utilized for documenting response. It is therefore essential to realise that oncological screening must be easily available, cost-effective, and time-consuming. Earlier detection of this short sequence protocol leads to prior and early breast cancer disease in high risky female populations like women with dense breasts, prehistoric evidence, etc. This proper utilization of AP reduces unnecessary mastectomies. Hence, this review focused on the explorative information for strongly suggesting the benefits of AP breast MRI compared to full diagnostic protocol MRI.

## 1. Introduction

Breast MRI plays a vital role in screening for breast cancer mainly because of its increased sensitiveness over mammography [1]. While considering the results in terms of biological activity and tumour architecture, the application of this method is particularly more useful for screening the complexities in women who are associated with

- high risk of developing breast cancer
- staging period evaluation
- follow-up after NAC (neoadjuvant chemotherapy)
- assessment of auxiliary lymph nodal region when the primary site could not be found through mammography

At present, breast MRI consumes about thirty to forty minutes according to the good practice suggested by benchmark protocols [2]. With this long screening time, the requirement for expertise for interpreting the results associated with huge direct and indirect costs limits the extensive utilization of breast MRI. Such huge cost is consumed for operation, acquisition, and maintenance of the equipment associated with expertise fees. This limitation led to the evolution of efforts for proposing an empirical selection of a limited number of acquisitions. Developing and enhancing such abbreviated MRI protocols will be effective both time and cost without negotiating accuracy [3]. Through a detailed investigation of the abbreviated ad, traditional MRI protocols for the detection of breast cancer, broader worldwide usage of this sensitive tool might be adopted in the future.

Shortening the high screening and complex protocol saves resources like hundreds of MRI room time hours. Meanwhile, the time for interpreting the results will decrease significantly with AP allowing innovative readability such as real-time and double reading interpretation. Further, more attention to image storage can be provided, which is crucial for the effective and efficient utilization of advanced imaging methodologies. Accordingly, the shorter examination could be better tolerated by the BC patients and decrease the patient motion that enhances the image quality.

## 2. Motivation for the Study

The review has been motivated by the urgent need and implementation of the most sensitive modality in the screening of BC. The utilization of breast MRI has been restricted because of the limited availability, high cost, high reading times, and long examination time. Hence, the review attracts the shorter MRI or abbreviated MRI protocols for BC screening. Recent findings state that AP decreased the actual handling time and time for evaluating the scan. These abbreviated scan protocols depicted the same sensitivity and nearly significant specificity compared to the FDP. For retaining the dynamic data, existing studies introduced ultrafast MRI that captures the contrast inflow in a lesion which is free with scan time. These results were also found to be stronger than the traditional curve types and could be exceptionally useful in the assessment of small lesions.

Further, this study has also been motivated by the hybridization or integration of shorter protocols to yield still more accurate results in reduced time with below 5 min scan time. The review also explores the limitations of the general studies like data biasing, limited samples that interpret a low number of cancers. Several studies reported that AP sensitivity is equal to the sensitivity of FDP. But specificity results seem to be heterogeneous and are found with a substantial decrease during the evaluation with breast AP [4].

## 3. Search Strategy

Table 1 represents the search strategy for this concise review. The search strategy has been processed with Google scholar, PubMed, and ScienceDirect, including the articles by standard publishers. The year of inclusion ranges from 2017 to 2021.

## 4. Breast Cancer Diagnosis

The objective of supplemental screening is to enhance the detection of early breast cancer in patients when conventional mammography has several limitations. With increased public awareness of increased breast density implications, most women prefer supplemental screening. But there exist uncertain and controversial suggestions regarding the vital impacts of supplementary screening in accordance to further reduction of BC mortality. Most of the national committee agrees that the predominant screening modality for dense breast women is routine mammography and has been depicted in Table 2 [5].

The challenges rely on the high NPV of the MRI and are characteristically utilized for the findings which are not benign but could not be sampled for biopsy [6]. Mammographic asymmetrical complexities, visible in one view, resulted in a negative MRI ruling out the malignancy [4]. In the meta-analysis, the absence of improvement at the calcification site has been correlated with an NPV of 93%. However, invasive cancer has been observed to be unlikely in this research setting. MRI outperforms galactography for nipple discharge with high sensitivity [7].

The traditional protocol used for Breast MRI is known as DCE-MRI which is short for dynamic contrast-enhanced MRI. It is used to assess the difference in neuroanatomical structures and kinetic information to be able to visualize breast lesions and be able to compare and distinguish malignant tumours from benign tumours. Some characteristics of a productive DCE-MRI are that it has a high spatial resolution through using thin slices (≤3 mm) and has a small pixel size (>1 mm) to enhance neuroanatomical features of lesions and increase the visualization of lobulated margins that are associated with malignant tumours, also considering high temporal resolutions (≤2 minutes); doing that supplies the lesion enhancement's kinetic details.

Considering breast DCE-MRI, there is not a standardized version like mammography solely because they are based on the radiologist's preference. However, it still includes the not upgraded fat-saturated T2 or STIR sequence to eliminate fat and asses fibroglandular tissues and be able to distinguish cysts from masses.

## 5. Abbreviated Protocol of Magnetic Resonance Imaging for Breast Cancer Screening in Dense Breast Tissue

Despite the fact that breast MRI has been observed to be a more sensitive examination in breast cancer detection, its utilization is constrained mainly due to its high cost [8]. Hence, some of the patients who are at a high risk of breast cancer could not adopt this cost-consuming screening process. Abbreviated protocol (AP) breast MRI is a better alternative and potential tool for screening with high accuracy and affordable cost. The following are the general studies that researched proving the significant outcomes of AP. [9] compared the interpretation time and diagnostic accuracy of AP over FDP of breast MRI using BI-RADS (Breast Imaging Reporting and Data System). This study also assessed the variability between the theoretical and practical classification of BI-RADS to gain information about diagnostic accuracy. This research has been conducted on 90 females who undergone 30 grade IV and V examinations which needs histopathological proof, 30 grade III examinations, and 30 benign examinations. Two radiologists were subjected to a review of the protocol independently. After preliminary analysis, the study found twenty-six cancers in 168 breasts. This study observed a high interpretation time for FDP protocol compared to AP. Accordingly, the classification reliability between both protocols is good. This paper perceived nearly 94.4% and 95.1% specificity and 100% sensitivity for FDP and AP, respectively. Meanwhile, [10] evaluated the utilization of AP with T2 weighted imaging in the detection of unifocal BC confirmed by biopsy. The radiologist assessed the MRI images according to T1 weighted images, T2 weighted images, and postcontrast images with prior history and imaging.

The framework has been compared for cancer detection, lesion conspicuity, and reading time. This study engaged a special breast radiologist for analysing the initial improvement in cancer ratio and for reviewing the final pathology and lesion morphology. A positive correlation exists between the initial enhancement ratio with increasing pathology and tumour grade. The lesion conspicuity increased, and the reader's interpretation time decreased when the initial enhancement ratio was increased. Finally, the paper concluded that AP possesses a high detection rate of breast cancer detection.

Similarly, FDP and AP were compared by [9] by a systematic meta-analysis that has been undertaken with multiple datasets. This analysis included cohort studies with accurate data in screening without enrichment from the moderate, high, and low levels of risk. The study made a comprehensive review of 23 articles where grade assessment reported very low significance between the specificity and sensitivity of both the AP and FDP methods. Meanwhile, this study suggested immense follow-up for the precise determination of clinical evaluation of the two methods.

Motivated by the fact that enhanced screening techniques have to be adopted for females with dense breasts due to their increased breast cancer risk and failed earlier diagnosis through mammographic screening, this paper [10] compared the performance of digital breast tomosynthesis and AP breast MRI in dense breast women. This longitudinal and cross-sectional study has been followed up to 48 sites in Germany and US from 2016 to 2017 within the age group of 40 to 75 years with routine screening. The study participants were subjected to both the screening methods in a randomized order, and independent reading was performed to prevent interpretation bias. This study focused primarily on the rate of invasive cancer detection. Secondary outcomes like specificity, sensitivity, recommendation rate, and positive predictive value were also included in this study. The research used pathology results of surgical biopsy as the reference standard. Among the dense breast women, AP compared with DBT has been correlated with a considerably higher detection rate of invasive breast cancer.

Accordingly, the research is required to understand better the relationship between the clinical outcome and screening methods. Consequently, [11] evaluated the use of AP comprising fat-suppressed T2 weight image analysis, post- and precontrast acquisition of the image and the subtracted max-intensity imaging for diagnosing women with BC surgery. This method performed AB-MRI examinations for 725 women with prehistorical surgery. Image acquisition time of 8.5 min has been obtained from this method. AB-MRI, ultrasound, and screening mammography are done around a similar time. Positive predictive value, cancer detection rate for biopsy and recall, specificity and sensitivity of MRI screening, and malignancy rate were evaluated. The study results suggested that this AP method could be adopted as the predominant screening modality.

The ultrafast-MR sequence before AP compared to FDP was investigated by [12] to differentiate the malignant lesions from benign. The study observed more specificity of 70.8% and 83% for FDP and AP with no considerable change insensitivity. With this ultrafast method, the interpreters could accurately diagnose 22.9% of malignancy and 10.4% of benign without missing abnormal cases. This study, in turn, depicted that both the interpretation time and image acquisition time are shorter compared to FDP.

## 6. Comparative Analysis of Traditional and Abbreviated Breast MRI Protocols

This section provides insights on the comparative analysis of FDP and AP breast MRI on breast cancer detection. This paper [13] depicted the reduced examination time with three minutes acquisition time and ten to fifteen minutes of occupation time followed by short reading time during the utilization of AP. In contrast, the acquisition time with FDP was thirty to sixty minutes, which will not permit more than two patients/hour in the diagnostic centre. This creates a path for increasing the breast MRI rate twofold with an intense indirect cost reduction. Further, this paper [13, 14] also proved that there is not much significant difference between the sensitiveness and specificity of AP and FDP results when analysing the reading time. AP takes 60 seconds for experts which is in contrast to the reading time of 120 seconds for FDP [15].

**Figure 1:**
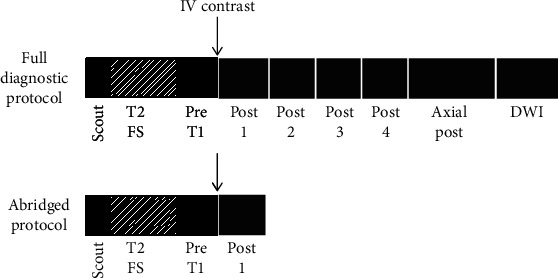
Shorter MR sequences of AP breast MRI protocol over FDP [16]

Figure 1 represents the MR shorter sequenced of AP breast MRI over FDP.

However, in [17], interpretation time has been longer than other existing methods because they did not utilize MIP images, and the interpretation was processed with a standard sequence. Further, in contrast to the general studies that utilized mere MIP images for AP interpretation, this paper did not perform MIP images since it has no impact on interpretation. So this study used subtracted images and native images for AP and FDP.

The utilization of late additive sequences for characterizing the lesion seems less clear since this study possesses a few cases like ACR 3 with AP and then reclassified as ACR2 with FDP. Further, this study found a specificity loss with AP despite the fact that late VIBRANT acquisitions were not utilized. This study possesses various limitations, like its retrospective nature. The readers were conscious of the indication, and 17 cases were already known. But other than tumour staging, cancer lesion detection was similar irrespective of examination. Accordingly, the identification rate matched the usual clinical circumstances for breast MRI interpretation. Further DCIS diagnosing remains a major challenge on MRI. In conclusion, this study deliberated that utilization of AP is thereby not detrimental in terms of interpretation reproducibility and care.

Similarly, [18] evaluated the use of AP of MRI compared to FDP of MRI in breast cancer screening within the dense breast. There are 478 female participants with negative mammographic results images with AP MRI and FDP MRI. The results were analysed separately, followed by the estimation of specificity and sensitivity. This study evaluated the ROC and chi-square test to assess the two protocols. Out of 16/478 cancer patients detected with FDP with nine ductal carcinomas, nine cases, six cases of invasive ductal carcinoma, and one mucinous carcinoma, ROC curves depicted the high efficiency of these adopted methods. There existed no statistical significance between the two methods [16].  Table 3 provides a comparison of AP with FDP for breast cancer detection.

## 7. Limitations

This section describes the major challenges and limitations enrolled in the existing studies. [16] found that this study could not be performed as just a screening study and designed as a reliability or feasibility test of AP and FDP in assessing the utilization of T2 weighted imaging and determining the exact type of cancers to be processed with this protocol [21]. Accordingly, during this research, the high-risk screening population with benign and negative examinations will also be supposed to calculate the specificity and sensitivity that consumes cost and time. Meanwhile, the radiologists were initially bounded with the history of the patients during the examination. These radiologists were aware that this series of breast cancer cases with associated biopsy clips enables readers to detect the cancer.

Hence, this limitation also increased detection rates associated with decreased interpretation time. This allowed the readers to stop reading the analysis immediately after the determination of the lesion. This study does not include maximum intensity projection images. Similarly, [11] also faced various limitations as follows: retrospective nature, single centred design, and considerably limited number of malignancies and patients. This study has not comprised all women with BC surgery history during this study period, which corresponds to selection bias. Further, the timing and number of previous screenings differed and did not affect the adverse outcomes of AB-MRI.

With the studies mentioned that have shown innovative results, they still had a restriction that affected the examination, having all the studies being a single institution. Most of them needed experts in radiology and being retroactive, obtaining only morphological characteristics of malignant tissues. There was a noticeable absence in the ability to assess kinetic information since there was a requirement for several timestamps postcontrast sequences to generate such information was removed. Previous studies [22] showed advanced features regarding breast cancer based on the patterns of the improvement difference regarding the delayed kinetic information stages, mainly the cancerous tissues. Reference [22] was the only study among the mentioned ones that did not exclude a nonfat-saturation sequence which may have influenced the results in evaluating the fat necrosis accurately [22]. A perfect example of an ideal method would have included an AP with both a scanning and a reading time and enable the use of assessing kinetic information.

Apart from that, relatively short verification bias occurs according to the cost-effectiveness, survival outcomes, and tolerability of the patients. This study promotes the preliminary data on utilizing AP-MRI in women with surgery history. This study strongly suggested the prospective and randomized multicentre study with a huge population for further evaluation of the findings. Precontrast and postcontrast subtraction images and MIP images in contrast to the previous images [23] were found to be not available in AP, which is the major limitation of the study. These subtraction images in the AP series could differentiate complicated cysts, bloody discharge, and enhanced lesions, whereas MIP enables the observers to evaluate the NME distribution. Further, this study did not compare the interpretation of previous studies. This study focuses on the interobserver variability of AP and the accuracy of AP MRI using an enriched cohort. Still, a slight decrease in the interobserver has been found. [12] also faced the limitations of retrospective centres with proven histopathological lesions that overestimated the cancer rate. Then, the scarcity of small cancers and the average tumour size of the lesions being more than 2 cm will limit the evaluation capability of the existing studies.

## 8. Critical Analysis

**Figure 2:**
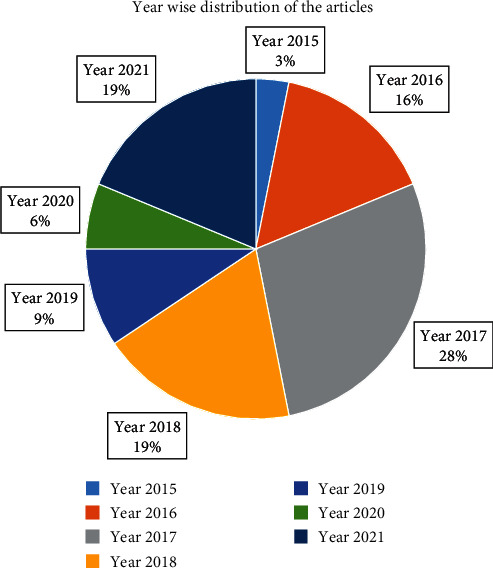
Year-wise distribution of the articles.

Figure 2 represents the year-wise distribution of articles involved in the exploration of possible suggestion in adopting AP for breast cancer detection.

## 9. Critical Discussion

The technical developments enhanced the breast quality MRI that allows high-resolution image acquisition. Additionally, the multiparametric breast MRI mainly replaced the conventional method for lesion classification. This allows for the estimation of a positive predictive value compared to mammography. Other investigated methods need a more quantitative MRI approach that increases the reproducibility and good consistency over the clinical area. Despite the consolidative results of breast MRI, there is a long-term exposure to gadolinium in BC patients undergoing frequent screening. The results of mammography nowadays become more questionable in the dense breast region. With an experienced team, MRI enables practical surgical improvement that reduces the re-excision, preventing nonreliable mastectomies.

Similarly, breast MRI enables the selection of patients to NAC and is functions as a modality in the therapeutic agent in assessing the residual size of the tumour [24]. After investigating the use of FDP in MRI screening, it is concluded that it has a long screening time and reading time, affecting the patient's ability to tolerate these examinations alongside the examinations being expensive. Enabling the use of decreased scanning MRI protocols can help decrease the examinations cost while still having its precision rate in diagnosing with increasing detecting rate of cancers which helps to have a faster exam and enables assessing breast MR scanning and cancer yield. Furthermore, using AP, there is no need to take more images, reducing storage system applications. Considering the absence of kinetic information ratio might hinder the understanding of malignant tissue identification.

Through the scanning studies that were conducted on patients that are at mild to moderate risk that did not show symptoms mammography wise, several malignant tissues were detected. These women are not allowed to undergo MRI screening as it has some restrictions to only be used for patients that are at high risk due to it being expensive, hence, the inability to be able to visualize and recognize these cancers until they are at a more advanced stage and start showing symptoms on patients. These results prove the importance of including women that are at lower risk in the eligibility criteria of MRI screenings which will then enable the use of AP [25].

The economic perspective in improving the patient's comforts could be optimized by adjusting the protocol that focuses on Abbreviated protocol [26]. Meanwhile, when a lesion has to be characterized or during high frequency, the multiparametric protocols are important. Since therapy should reduce the breast lesion enhancement, proper and reduced time evaluation with AP-breast MRI must be adopted.

## 10. Conclusion

The high cost of the examination has constrained breast MRI as a screening tool. To adopt more widespread utilization of this sensitive screening method, it is significant to decrease the examination price and scanning period. The practical approach involves reducing acquisition time that allows for increased patient throughput, which probably increases the expansion and acceptance of the screening population. This, in turn, significantly reduce the intermediate disease development risk in women with extremely dense breast and a family history with no hereditary mutation. However, the specificity of AP is found to be slightly lower than FDP, leading to a high recall rate and additional assessment. This review strongly suggested that ultrafast or abbreviated protocols or the combined effect will be a better alternative to FDP in terms of acquisition time, examination time, cost, and availability.

In conclusion, the review suggested that fast sequences or AP breast MRI correlated with a short imaging period might reduce the interpretation cost and time. It also maintains the accuracy of the diagnosis, which is similar to FDP. This comprehensive review suggested that the reliability and application of AP should be adopted, organised in a huge prospective series.

## Figures and Tables

**Table 1:** 

Keywords	Source	Years
(i) Cancer (ii) Breast cancer (iii) Significance of breast cancer detection (iv) Modern technological protocol in breast cancer detection (v) MRI for breast cancer detection (vi) Full diagnostic protocol (vii) Abbreviated protocol (viii) Advantages of abbreviated protocol (ix) Recent trends in AP and FDP (x) Screening modalities (xi) Parameters involved in breast cancer detection	(i) Articles identified through Google Scholar searching from 2017 to 2021 (ii) Additional records were obtained through PubMed and ScienceDirect database from 2017 to 2021	2017 to 2021

**Table 2:** The screening modality for dense breast women is routine mammography.

S. no	Year	National Group	Suggestions on supplementary screening	Suggestions on primary screening
1	2016	USPSTF (United States Preventive Service Task Force)	Digital mammography reveals good results for younger women and dense breast possessing women	Recent evidence is insufficient for assessing the balance between the merits and demerits of adjuvant screening with MRI, ultrasonography and other methods
2	2013	ACS (American Cancer Society)	Although overall sensitivity is similar for FDP and AP, digital mammography reveals good results for younger women and women with dense breasts	Data accumulation on breast tomosynthesis seems to provide better results with improved accuracy
3	2015	ACR (American College of Radiology)	Annual mammogram screening is represented for intermediate and high-risk women. In addition, CE MRI is represented, and ultrasound may be regarded as the alternative to clarify the MRI's contrary indications	Mammography with supplementary screening always derived more consecutive results in dealing with predispositions of dense breast women and to provide accurate detection of results
4	2009/2016	NCNN (National Comprehensive Cancer Network)	Digital mammography benefits younger women and women who have dense breast	Despite few studies supporting the utilization of ultrasound scans for BC screening in women with high risk, the NCCN panel warns that there is inadequate proof for supporting routine screening in dense breast women associated with no other observed risk factors

**Table 3:** Comparison of AP with FDP for the detection of breast cancer.

S. no	Author	Number of breast cancer observed/total number of investigated cases	Abbreviated protocol	Duration of acquisition time	FDP breast MRI		AP breast MRI	
					Specificity	Sensitivity	Specificity	Sensitivity
1	[19]	11/678 (screening in high risk population)	T1 pre- and postcontrast imaging	3 minutes	97.4%	81.8%	97.2%	81.8%
2	[20]	110/180 (screening in only lesions)	T1 pre- and postcontrast imaging	7 minutes	95%	97%	93%	99%
3	[21]	14/356 (screening in dense breast)	T1 pre- and postcontrast imaging and DWI	6 minutes	96.8%	100%	95%	100%
4	[22]	207/508 (screening of clinical population)	T1 pre- and postcontrast imaging	3 minutes	77.1%	99.5%	75.4%	99.5%
5	[23]	16/478 (screening in dense breast)	T1 pre- and postcontrast imaging	3 minutes	94.6%	100%	88.3%	93.8%
6	[24]	75/470 (screening of clinical population)	T1 pre and post contrast imaging, T2 STIR and T2	10 minutes	92%	92%	91%	89%
7	[10]	107 cancer population	T1 pre- and postcontrast imaging	7 minutes	—	99.4%	—	99.4%
8	[25]	7/568 (screening population)	T1 pre- and postcontrast imaging	4.4 minutes	96%	100%	94%	100%

## Data Availability

The data that support the findings of this study are openly available in reference number.
